# The use of nanomaterials as drug delivery systems and anticancer agents in the treatment of triple-negative breast cancer: an updated review (year 2005 to date)

**DOI:** 10.1186/s11671-024-04089-3

**Published:** 2024-09-03

**Authors:** Tanaka Ndongwe, Angel-Alberta Zhou, Nelisa Paidamwoyo Ganga, Nyaradzo Matawo, Unami Sibanda, Tinotenda Vanessa Chidziwa, Bwalya A. Witika, Rui W. M. Krause, Gauta Gold Matlou, Xavier Siwe-Noundou

**Affiliations:** 1 Department of Pharmaceutical Sciences, School of Pharmacy, Sefako Makgatho Health Sciences University, Pretoria, South Africa; 2 Department of Pharmacy, School of Health Science, University of KwaZulu Natal, Durban, South Africa; 3 Department of Pharmacy and Pharmacology, Faculty of Health Sciences, University of the Witwatersrand, Johannesburg, South Africa; 4 Pharmaceutics Division, Faculty of Pharmacy, Rhodes University, Grahamstown, South Africa; 5 Chemistry Department, Faculty of Science, Rhodes University, Grahamstown, South Africa; 6 Electron Microscopy Unit, Sefako Makgatho Health Sciences University, Pretoria, South Africa

**Keywords:** Triple negative breast cancer, Nanomaterials, Drug delivery systems

## Abstract

Triple-negative breast cancer (TNBC) is characterised by the lack or low expression of estrogen, progesterone, and human epidermal growth factor receptor 2 receptors. TNBC has a high recurrence rate, swiftly metastasizes, and has a high mortality rate. Subsequently, the increase in cases of TNBC has signaled the need for treatment strategies with improved drug delivery systems. New diagnostic approaches, chemical entities, formulations particular those in the nanometric range have emerged after extensive scientific research as alternative strategies for TNBC treatment. As compared to contemporary cancer therapy, nanoparticles offer peculiar tunable features namely small size, shape, electrical charge, magnetic and fluorescent properties. Specifically in targeted drug delivery, nanoparticles have been demonstrated to be highly efficient in encapsulating, functionalization, and conjugation. Presently, nanoparticles have ignited and transformed the approach in photodynamic therapy, bioimaging, use of theranostics and precision medicine delivery in breast cancer. Correspondingly, recent years have witnessed a drastic rise in literature pertaining to treatment of TNBC using nanomaterials. Subsequently, this manuscript aims to present a state-of-the-art of nanomaterials advance on TNBC treatment; the ubiquitous utility use of nanomaterials such as liposomes, dendrimers, solid lipid nanomaterials, gold nanomaterials and quantum dots as anticancer agents and drug delivery systems in TNBC.

## Introduction

Among the various types of breast cancer, triple-negative breast cancer (TNBC) has become a significant global concern due to its notably high mortality rate [1]. The clinical diagnosis of TNBC is confirmed by the absence of estrogen receptor (ER), progesterone receptor (PR), and human epidermal growth factor receptor type 2 (HER2) expression [2]. Literature suggests that TNBC is predominantly diagnosed in women of color under the age of 50, specifically African or Hispanic descent, and accounts for 10–20% of all reported cases of invasive breast cancer [3]. The molecular profile of TNBC reveals multiple subclasses, including basal-like tumors and normal breast-like tumors [4]. Common clinical features of TNBC include high mortality rates, recurrence rates, and metastases which makes it one of the most dreaded cancer types [5].

Current treatment strategies for TNBC are primarily chemotherapy, radiation, and surgery [6]. However, these strategies present several limitations, including off target therapy, heterogeneity and interactions with microenvironments [1]. In addition, these tumors are often associated with resistance to conventional therapies, particularly multifactorial resistance mechanisms which include genetic alterations, microenvironmental changes, and cancer stem cells [1, 7]. Despite the continuous use of chemotherapy as the primary systemic treatment in TNBC patients with a commendable of clinical response, poor prognosis and high risk of relapse and reoccurrence are a recurring concern [8]. This complexity of the issue is further increased by the inability of hormone therapy to effectively treat TNBC [9]. In recent years, nanomaterials have emerged as a promising avenue for TNBC therapy, offering novel anticancer agents and delivery systems to potentially overcome the limitations of conventional treatment.

Nanomaterials have shown potential to bridge the therapeutic gap in TNBC treatment and offer tremendous advantages in formulating anticancer agents or ideal drug delivery systems as they possess unique versatile functionalities [10]. A number of nanoparticle formulations have demonstrated the ability to penetrate leaky vasculature associated with tumors and heterogeneity surrounding tumors [11]. Moreover, nanomaterials have the ability to enhance deposition of nanomedicines precisely to the site of disease through drug release mechanisms triggered by factors like pH, redox potential, enzyme presence, or temperature. Furthermore, the site specific targeting of nanomaterials also address tumor microenvironments (TME) and the vessel extracellular matrix to prevent spreading (proliferation), reduce off target toxicity, enhance drug uptake and drug deposition in the cancerous tissues [12, 13].It is well known that tumor cells are usually surrounded by several other cells including immune cells, fibroblasts, lymphocytes, growth factors, tumor vasculature, and proteins forming the TME [14]. In the TME, rapid tumor growth and irregular blood vessels cause low oxygen levels (hypoxia). This hypoxia leads to a weakened immune response by inhibiting dendritic cell functions and causing abnormal cells resulting in fibrous tissue growth. Nanoparticles can be engineered to target and modulate the TME by reducing the number of immunosuppressive cells or transforming the TME from immune-suppressive to immune-supportive [7]. Furthermore, the capacity of nanomaterials to engage at both molecular and cellular levels creates new opportunities for the development of novel anticancer therapies.

Nanomaterials have been demonstrated to improve drug solubility, targeting cancer cells, reduce side effects and enhance drug entrapment efficiency. As a result, nanomaterials may circumvent the shortcomings of chemotherapy such as poor solubility, and devastating side effects. Lipid, polymeric, quantum, carbon, and gold nanoparticles are some of the leading examples of nanomaterials that have repeatedly been found as promising anticancer agents and drug delivery systems. Particularly, in TNBC, lipidic nanomaterials have demonstrated considerable improvements in the therapeutic efficacy, toxicity, and treatment resistance of anticancer drugs [15]. Polymeric nanomaterials have been recognized as versatile drug delivery systems that enhance the bioavailability of anticancer agents, reduce drug toxicity, and improve the targeting of these agents [16]. Additionally, other nanomaterials, including quantum dots have set a tone for disease diagnosis and treatment. Carbon-based stimuli-responsive materials offer numerous advantages in clinical settings as they are effective promising drug delivery systems, biosensors agents and imaging agents [17, 18]. Other significant advances that have been made possible is the drug delivery of anticancer agents through the application of hydrogels in conjunction with magnetic microrobots [19]. Quantum dots, carbon and gold nanoparticles, have been highlighted as potential biosensors and imaging agents in cancer therapy [20]. These nanoparticles not only inhibit cell proliferation and progression but also have the potential to prevent further metastasis [22–24].

Given the notable and devastating side effects associated with the current treatment strategies for TNBC more alternatives with ideal safety, efficacy, and profile for TNBC are thus needed [25]. As a result, this review presents the promising role of nanomaterials in TNBC as anticancer agents and drug delivery systems. Advances regarding the leading nanomaterials and how they circumvent the shortcomings associated with the current TNBC therapy are also underscored. The articles considered in this manuscript were published between the years 2005 to date.

### Current treatment of TNBC

**Fig. 1:**
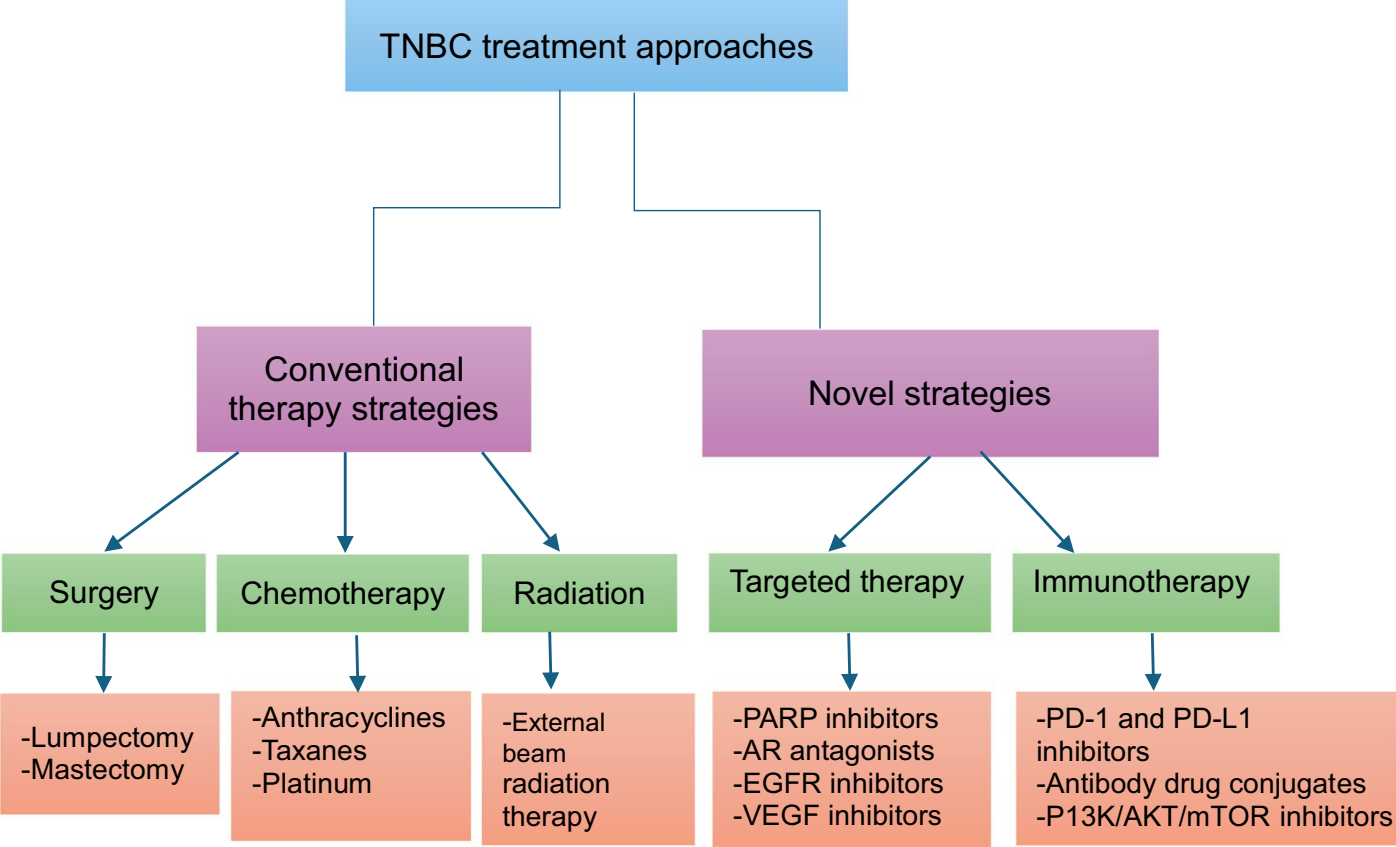
TNBC treatment approaches [6]

Triple negative breast cancer lacks ER/PR receptors together with the lack of overexpression of HER like in all the other breast cancers subtypes [26]. While this makes TNBC a distinct molecular subtype, it can further be categorized based on specific molecular subtype. The subcategories can help identify treatment options however, the distinctions are not well defined, and patients may present with one or more TNBC subtype. Targeted therapy for TNBC patients is currently under investigation. Therefore, chemotherapy has been the only active treatment available as shown in Fig. 1 and Table 1 [26, 27]. In this respect, TNBC tumors also show heterogeneity, which has implications on treatment [27, 28].

**Table 1:** Treatments commonly used in TNBC

Class	Mechanism of action	Example(s)	Common side effect(s)	Precautions
Taxanes	Act by disrupting the microtubular network in cells	Docetaxel Paclitaxel	Risk of fluid retention Gastrointestinal side effects	Pretreat the patient with steroids [29]
Halichondrin	Inhibits the growth phase of microtubules alternately leading to apoptotic cell death	Eribulin	Neutropenia, alopecia, global peripheral neuropathy	Adjust dose in liver or kidney dysfunction Possible irreversible infertility in male patients [29]
Epothilones	Stabilizes the dynamics of microtubules, which causes blockade of cancer cells during the mitotic stage of cell division cycle. This eventually results in apoptosis and cell death	Ixabepilone	Sensory neuropathy	Hypersensitivity [29, 30]
Anthracycline	Disrupt nucleic acid synthesis, thereby causing growth arrest and programmed cell death	Doxorubicin (Dox)	Alopecia, Gastrointestinal, and neutropenia side effects	Safe and mild to moderate hepatic dysfunction [6]

### Advances in TNBC treatments

The limitations associated with the conventional TNBC treatments have led to the development of novel treatment strategies as highlighted in Fig. 1. Examples include immunotherapy, antibody–drug conjugates and PARP inhibitors. TNBC tumors harbor basal like phenotypes which are seen on immunohistochemical staining of the tumor slides during diagnosis [29]. These are basal-like types one and two, immunomodulatory, mesenchymal-like, mesenchymal stem like, and luminal androgen receptor. TNBC tumors also differ in their gene expression and respond to chemotherapy and biological therapy differently. The choice of treatment selection, therefore, will depend on various factors. Evaluation of patient-by-patient case of the biopsy results to show the subtype of triple negative breast cancer is essential. Factors such as whether the metastasis cancer is present and the site of metastasis, assessment of the patient’s Eastern Cooperative Oncology Group performance status, tumor burden, rate of progression, organ involvement and function as well as residual toxicities from prior therapy need to be evaluated [29]. The specific agent to be used for treatment is also dependent on whether the patient has received treatment previously [29]. For patients who have previously received treatment, but with TNBC progression, resistance is assumed and a different agent with a different mechanism from the previous one is used [26, 29]. Patient's choice may play a role, as some patients would prefer more aggressive and effective treatment whereas others will avoid serious side effects and opt for lesser aggressive treatment [26, 29].

Each treatment option for TNBC has its own set of challenges which can vary on the characteristics of the tumor and the patient. Programmed death-ligand 1 (PD-L1) positive tumor cells can be susceptible to immune checkpoint inhibitors such as pembrolizumab and atezolizumab which improve outcomes; however, the cells are prone to immunotherapy resistance and not all patients respond to these therapies. For TNBC cancer cells with either BRCA1 or 2 gene mutation, patients may benefit from poly (ADP-ribose) polymerase (PARP) inhibitors like olaparib. Specifically, because they inhibit DNA repair mechanisms in cancer cells with BRCA mutations. However, cancer cells may acquire more resistant mechanisms to bypass the PARP inhibitors. Chemotherapy depicted in Table 1 is often used in combination with these targeted therapies which is beneficiary to cancer that does not express either BRCA mutant or PD-L1 [29, 30].

#### Immunotherapy

Immunotherapy using check-point inhibitors coupled with neoadjuvant chemotherapy has shown to be a relevant strategy for treatment of TNBC cells that are highly immunogenic [31]. Antibodies targeting the programmed cell death protein 1 PD-1 and programmed cell death ligand 1 PD-L1 inhibitor receptors such as pembrolizumab and atezolizumab have been approved by the FDA for treatment of TNBC [31]. Of interest is the Phase Ib KEYNOTE-012 trial conducted by Nanda et al. [32] which evaluated the anti-tumor efficacy and safety profile of pembrolizumab. The preliminary results supported further development of pembrolizumab for treatment of metastatic TNBC, the overall response rate was 18.5% [33]. Also, a phase 3 trial by Cortes et al. [34], compared the combination of pembrolizumab and chemotherapy versus chemotherapy alone. The results from this study proved that the combination of pembrolizumab and chemotherapy had a significant impact on the overall survival of the TNBC patients [34].

#### Antibody drug conjugates

Antibody drug conjugates (ADCs) have shown great promise in the treatment of cancer. An ADC is comprised of the following: antibody targeting a tumor antigen, cytotoxic payload and a linker between the antibody and payload [35]. Sacituzumab govitecan, is an ADC approved by the FDA in February 2023 for metastatic TNBC [36]. The approval of sacituzumab govitecan is supported by the results from a phase 3 TROPiCS-02 study conducted in 2022. Rugo et al. [39] reported that sacituzumab govitecan had significant clinical efficacy in the treatment of heavily pre-treated HR + /HER2− endocrine-resistant, unresectable locally advanced or metastatic breast cancer patients.

#### PARP inhibitors

Poly (ADP-ribose) polymerase 1 (PARP1) is an important protein that facilitates DNA repair. PARP1 is overexpressed in breast cancer tumors involving BReast CAncer gene 1 (BRCA1) or BReast CAncer gene 2 (BRCA2) mutation. The inhibition of PARP1 and Poly (ADP-ribose) polymerase 2 (PARP2) leads to inactivation of cells that lack functional BRCA1 or BRCA2 resulting in increased genomic instability and cell death [37, 38]. Two PARP inhibitors, olaparib and talazoparib have been approved for treatment of metastatic TNBC. Other PARP inhibitors currently under clinical investigation include talazoparib, rucaparib, and veliparib [40].

### Shift from conventional dosage form to nanomaterials in TNBC

Over the years, the treatment of TNBC has witnessed a paradigm shift from conventional to novel approach through the intrusion of nanotechnology. Of notable interest is the recent development of nanometric formulations which can precisely deliver the drugs through active and passive targeting sites. Nanomaterials that are under immense investigation in TNBC include but certainly not limited to polymeric nanoparticles, metallic nanoparticles, inorganic nanoparticles and carbon nanomaterials [41]. To date a commendable of anticancer nano formulations have been approved by the FDA such as liposomal doxorubicin (PEGylated), liposomal doxorubicin (non-PEGylated) and Albumin-bound nanoparticles with paclitaxel [13]. In addition to this, preclinical studies are also exploring the combinatorial use of nanotechnology in formulation of chemotherapeutic agent’s delivery with free immunotherapeutic drugs.

### Clinical aspects of nanomaterials and the shift

The current prominent treatment strategies of TNBC are surgery and chemotherapy. Notwithstanding, their continual usage relapse, toxicity and poor prognosis have been recurrently linked to the aforementioned approaches. The formulation of drugs using nanotechnology implicated in the treatment of TNBC has proven to be effective in TNBC therapy. Leading anticancer drugs including but certainly not limited to paclitaxel which has been formulated to nab paclitaxel a nanomaterial which demonstrated efficacy [42, 43]. Nanotechnology strategies also necessitate the visualization of drugs at the target site through the use of co‐delivery of therapeutic and imaging agents allowing both treatment and bioimaging [44]. Furthermore, improved targeting of proteins implicated in TNBC cancer such as folate receptor, Urokinase Plasminogen Activator Receptor, Epidermal Growth Factor Receptor, Insulin Growth Factor 1 Receptor, Wnt Pathway, Mucin 1 (MUC1), Folate Receptor and C-X-C chemokine receptor 4 (CXCR4) [45].

## Nanoparticles use in the treatment of TNBC

Given the distinctive characteristics of tumor cells in TNBC, strategies for effective treatment are required. Despite the availability of targeted therapies for PD-L1 positive and BRCA mutant subtypes, chemotherapy is still recommended and frequently used [46]. The main challenge in the use of chemotherapy lies in precise targeting of the cancer and immune cells in TNBC patients. Additionally, TNBC patients often encounter resistance to chemotherapy as compared to other breast cancer types [46, 47]. Nanomaterial use emerges as a promising method to overcome some of these challenges in TNBC treatment [46]. Nanomaterials are drug carriers and therapeutic agents that facilitate drug delivery to specific targets often use temperature conditions around the tumor cells [47]. The use of nanomaterials in TNBC can be attributed to their unique properties which include high retention in the circulatory system, large surface area-to-volume ratio which can be further modified with ligands for tumor targeting, increased preferential permeability in tumor tissues and high encapsulation for targeted drug delivery, which protects the drug from degradation and premature release before the target site [31, 48, 49].

### Targeted drug delivery based on physical stimuli

**Fig. 2:**
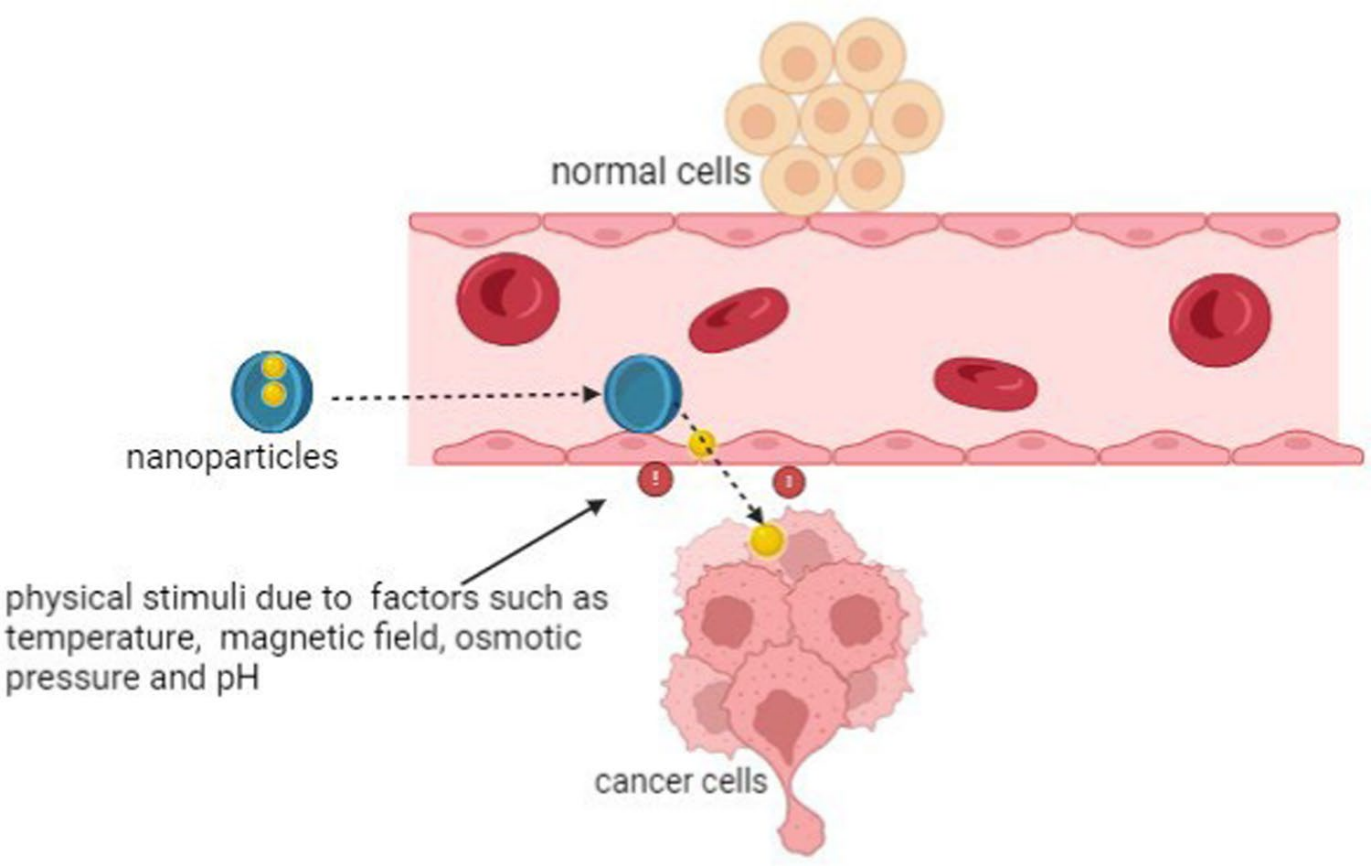
Physical stimuli of nanoparticles [55]

The effectiveness of nanomaterials in targeting TNBC cells is contingent upon their ability to release the drug in reaction to different stimuli. Stimuli based delivery offers controlled and sustained drug delivery releasing the drug based on external or internal stimuli, primarily to minimize systemic side effects enhancing treatment efficacy (Fig. 2) [8]. Magnetic fields with frequencies below 400 Hz are minimally absorbed by tissue, allowing remote manipulation without direct physical or chemical interaction. Magnetic nanoparticles, which respond to magnetic fields, can be used for various applications, including magnetic targeting for precise drug delivery, magnetically triggered release of drugs [50]. Thermo-responsive nanocarriers, made from temperature-sensitive polymers, release drugs in response to temperature changes, reducing off-target toxicity. These carriers release their payload at target tissues after hyperthermic stimuli (37–42 °C) to avoid protein denaturation and tissue damage [51]. Ultrasound has also become prominent in targeted and responsive drug delivery, triggering the release of active molecules from polymer matrices via regional sonication. This method, which uses ultrasound to release contrast agents from nanocarriers with the help of microbubbles, is a well-established diagnostic technique that enhances uniform drug distribution and tumor uptake [52]. The use of light as an external stimulus offers several benefits, such as ease of application, biocompatibility, and precise spatial and temporal control. Encapsulated or conjugated bioactive agents are released when exposed to light of specific frequencies, such as UV, near IR, and IR. These frequencies are compatible with tissue and potent enough to induce conformational changes in the chemical structures of nanocarriers [53, 54].

**Fig. 3:**
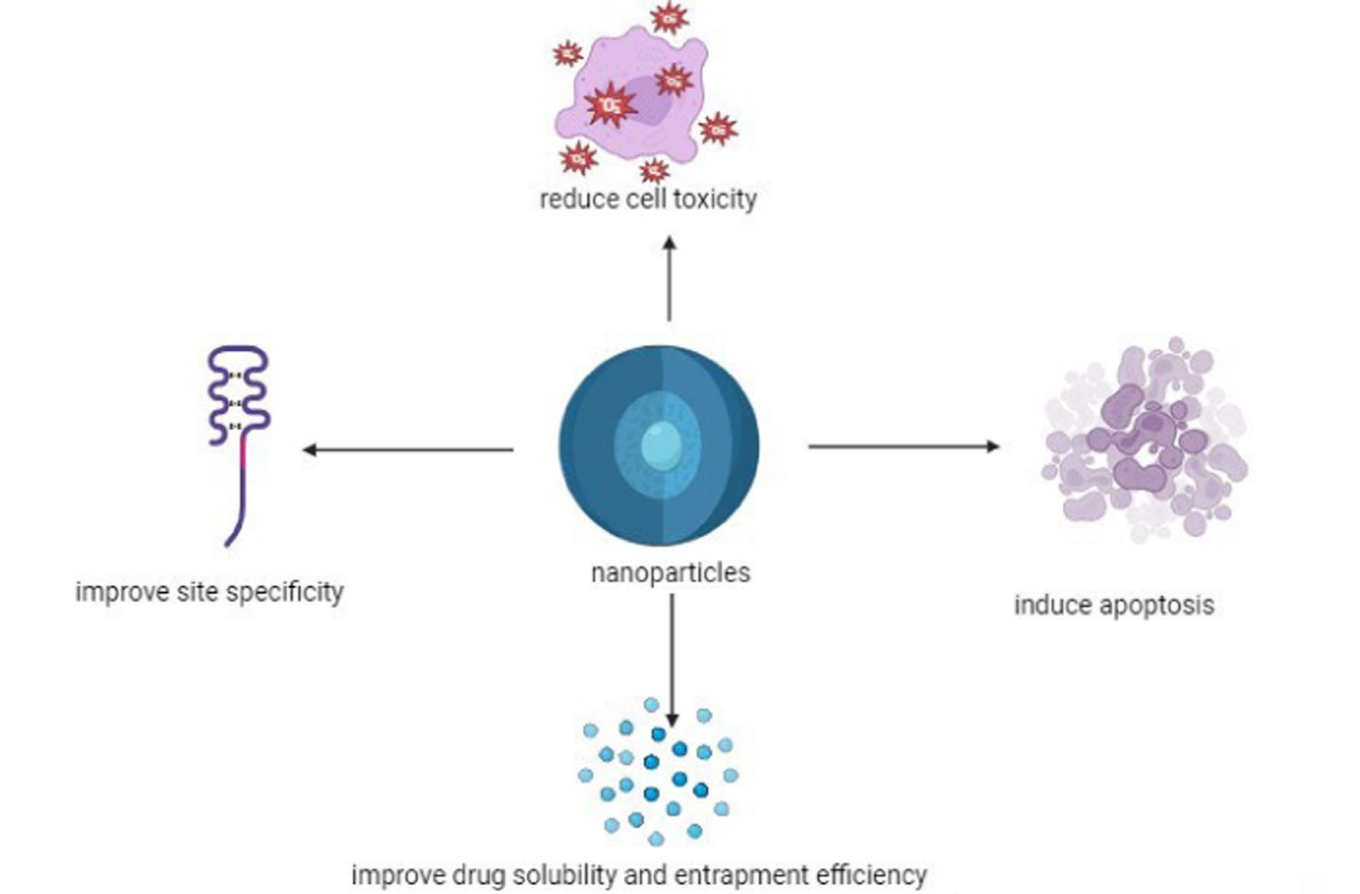
Role of nanomaterials in TNBC therapy [56, 57]

These properties collectively overcome the limitations of conventional treatment, such as poor solubility and low bioavailability. Figure 3 highlights the benefits of the nanomaterials discussed in the sections below, which contribute to better outcomes and reduce systemic toxicity in patients.

### Nanomaterials as drug delivery systems

#### Liposomes

**Fig. 4:**
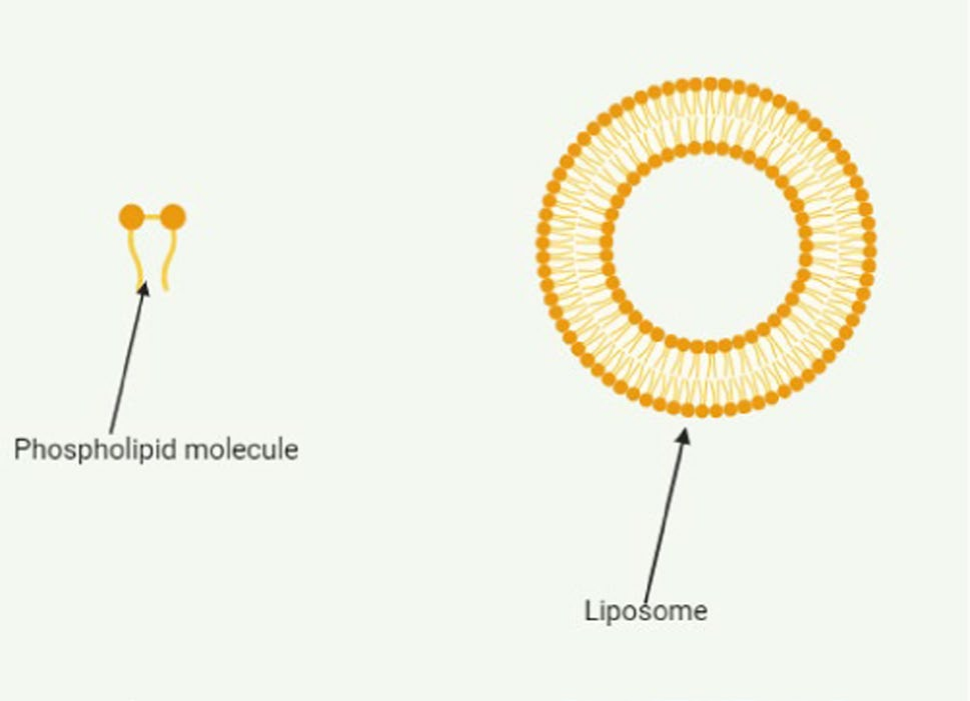
Liposomes as drug delivery systems

Liposomes are spherical vesicles (Fig. 4) that are nanosized (100–400 nm) and are made up of phospholipid bilayers surrounding an aqueous core. In drug delivery, liposomes can encapsulate both hydrophobic and hydrophilic drug molecules. Liposomes are appealing due to their non-immunogenic properties, potential to reduce systemic toxicity, and ability to improve drug efficacy [31, 58]. For instance, Doxil® an anthracycline-type chemotherapy drug used in TNBC therapy form doxorubicin (DOX) has been reported to have reduced systematic and cardio toxicity as compared to the free doxorubicin drug. The liposomal formulation of DOX was discovered to minimize side effects as is now predominantly used in TNBC therapy [58, 59].

In a research study by Chen et al. [59] gadolinium (Gd) and DOX loaded within polyepoly(ethylene glycol)-poly(ε-caprolactone) (PEG-PCL) copolymer nanomaterials modified with anti-intercellular adhesion molecule 1 (ICAM1) was synthesized and characterized. The synthesis was achieved using the double emulsification of PEG/PCL with a combination of DOX and Gd and subsequent modification with anti-ICAM1. The formulation inhibited the proliferation of TNBC (MDA-MB -231) cells with an inhibitory concentration (IC_50_) of 0.29 µg/mL when tested for their *in-vitro* cytotoxicity. The synthesized nanoparticle delivery system demonstrated enhanced diagnostic and therapeutically efficacy by specific targeting ability and extended circulation time in the tumor models. The proposed approach can be used for MRI guided therapy in TNBC [59].

Dai et al. prepared and characterized doxorubicin-loaded LXY-modified stealth liposomes (LXY-LS) and rapamycin (RAPA) (loaded micelles (M-RAPA) for targeting integrin a3 to combat TNBC. The findings show that LXY modification significantly enhanced the cellular uptake of liposomal DOX in integrin a3 overexpressed. Furthermore, in vitro cytotoxicity effect of DOX and RAPA against model TNBC cells showed IC_50_ of 3.09 µg/mL and IC_50_ of 0.89 ug/ml, respectively, showing synergy of DOX and RAPA. Additionally, in vivo anti-tumor activity against mouse bearing TNBC show a decrease in relative tumor volume compared to the control from 3.36 to 1.63. According to the findings, targeted combinational therapy based on LXY-LS-DOX and M-RAPA systems may offer a rational strategy to enhance therapeutic outcomes of TNBC [60].

Liposomes have been studied not only in the field of chemotherapy, but also in photodynamic treatment (PDT). Liposomes can transport photosensitizers to tumors, which can cause cytotoxicity when activated by light of specific wavelengths [31]. Shen et al. developed a liposome-based ruthenium polypyridine complex, (ClO4)2 (Lipo-Ru), which emits a high fluorescent signal when incorporated into the hydrophobic lipid bilayer for the delivery vehicle or into the DNA helix, enabling visualization using confocal microscopy to monitor therapeutic agent in tumor tissues. Lipo-Ru significantly showed cell apoptosis of approximately 75.9%. According to the findings of the study, Lipo-Ru could be a promising platform for TNBC therapy [61].

Shemesh et al. [62] enveloped and assessed a liposomal nanodelivery system utilizing indocyanine green (ICG) as a photosensitizer, activated by near-infrared (NIR) irradiation, for in vivo photodynamic therapy (PDT) in TNBC. The study results demonstrated a significant 96% reduction in cell viability upon photoactivation of liposomal ICG with NIR radiation. Additionally, NIR fluorescence imaging showed enhanced tumor accumulation. The nano-based liposomal ICG has the potential to offer pharmacological effects and real-time formulation monitoring through their fluorescent properties [62].

Another class of interest is the recent use of polymer including coordination polymers (CPs) which can be synthesized using solvent free methods. The broad application of CPs not only extends to sensors but also as drug delivery systems [63]. Zirconium (Zr^4^^+^)-containing nanoscale coordination polymers were successfully prepared by Li et al. [64] using Zr^4^^+^ as metal ion nodes while tetrakis(4-carboxyphenyl) ethylene was utilised as ligands. Functionalized pyrene-derived polyethylene glycol (Py-PAA-PEG-Mal) was used for surface modification of the coordination polymer, and further conjugated with cRGD. The study's findings showed that functionalized formulation demonstrated an excellent capability for tumour cell targeting imaging based on cellular evaluation in TNBC.

Li et al. [65] used CPs to design a formulation that use combinatorial therapy. The researchers developed nanoscale coordination polymer nanomaterials containing carboplatin (carb) in the core and the photosensitizer pyrolipid (pyro) on the shell to effectively treat metastatic TNBC. Upon exposure to UV radiation, the formulation demonstrated the release of reactive oxygen species. When administered through intravenous injection and subjected to local light irradiation, carb/pyro exhibited a significant regression in tumor growth within the 4T1 murine metastatic breast cancer model. Furthermore, the formulation, functionalized with anti-CD47 antibody, carb/pyro, combined with light irradiation, resulted in the complete eradication of primary and metastatic 4T1 tumors in 50% of the mice.

#### Solid lipid nanomaterials

**Fig. 5:**
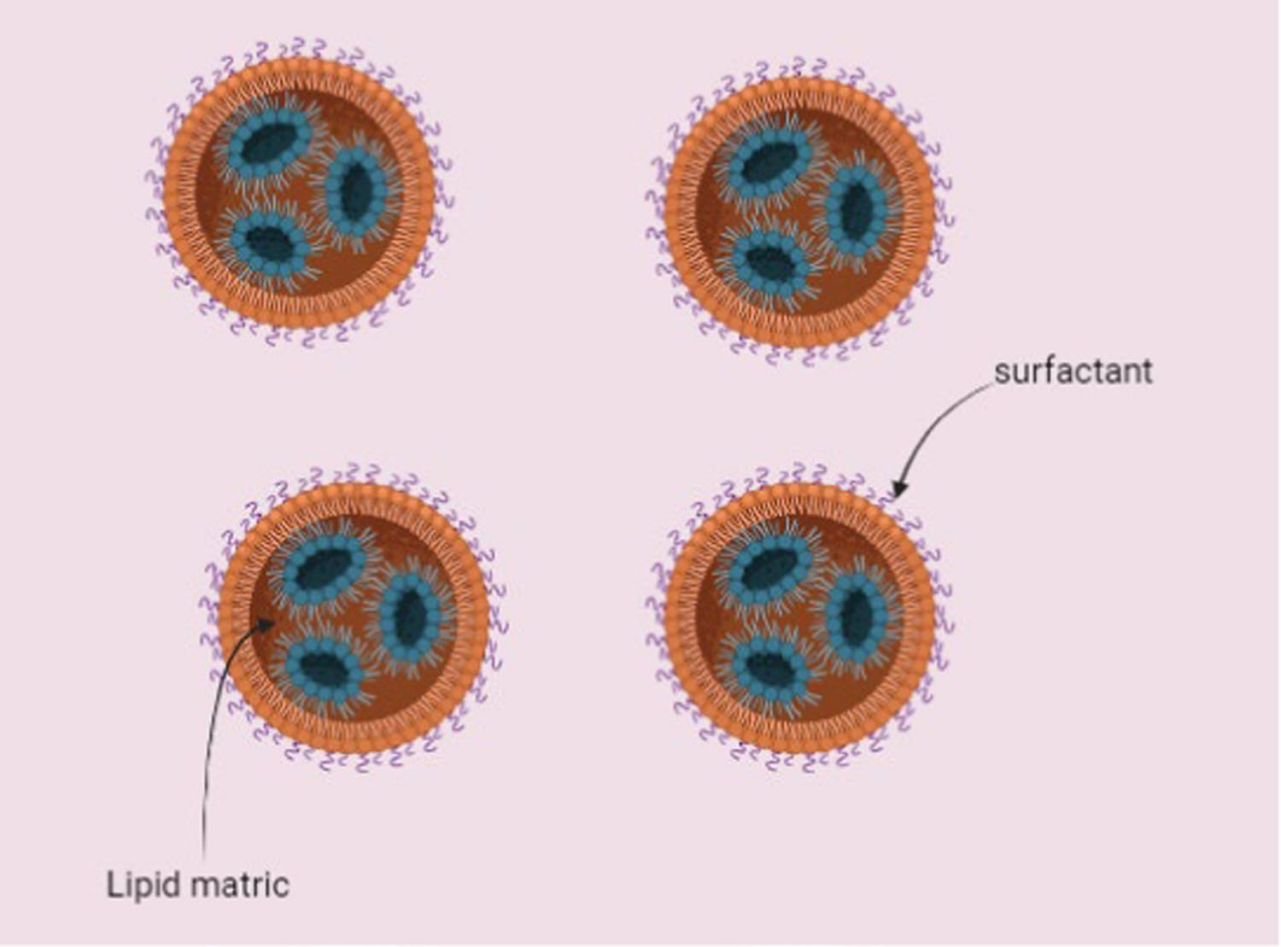
Solid lipid nanoparticles used in drug delivery

Solid lipid nanomaterials (SLNs) consist of biodegradable lipids and exhibit a spherical structure with sizes typically falling within the range of approximately 100–1000 nm [31]. These nanosized particles shown in Fig. 5 possess versatility and utilized in both therapeutic and diagnostic applications. The absence of organic solvents during their preparation grants SLNs a unique advantage over other delivery systems [3]. Research on SLNs in cancer treatment has demonstrated their ability to effectively address the challenges of multi-drug resistance encountered in cancer therapy [66].

Scialla et al. [67] conducted a study with the objective of designing a magnetic hybrid nanovehicle for targeted treatment of TNBC. The nanovehicle aimed to achieve pH-triggered targeting of tumor-associated macrophages (TAMs), offering localized imaging and therapeutic potential with enhanced safety. In vitro and in vivo studies were performed to characterize the prepared formulation using the MBA-MB-231 TNBC cell line and a TNBC mouse model with M-Wnt tumors, respectively. The investigation results indicated that mice treated with AS_mLNVs-DOX experienced significantly higher tumor growth inhibition compared to mice treated with the free drug (DOX), resulting in a 58% reduction in tumor volume. Moreover, the delivery system improved safety and drastically reduced adverse effects. Unlike the free drug (DOX) that induced necrosis at the injection site, no toxic effects were observed for AS_mLNVs-DOX upon visual inspection. The proposed solid lipid nanovehicle delivery system shows great potential in providing effective and safe delivery of DOX for TNBC treatment. It offers the benefits of targeted therapy, localized imaging, and minimized adverse effects [67].

In another study, Siddhartha et al. [68] developed solid lipid nanomaterials (SLNs) loaded with di-allyl-disulfide (DADS) and engineered them with a receptor for advanced glycation end products (RAGE) for the treatment of triple-negative breast cancer (TNBC). DADS acted as the cytotoxic agent to overcome issues with bioavailability, while RAGE served as a target to deliver the drugs specifically to TNBC cells. The study reported a remarkable 61.8% antitumor activity, demonstrating the efficacy of DADS-RAGE-SLN in combatting TNBC. Furthermore, the cytotoxic effects were effectively reduced, indicating the potential of this approach in minimizing off-site targeting effects. DADS-RAGE-SLN presents a promising strategy for antitumor activity, specifically targeting TNBC cells, and mitigating side effects on non-target tissues [68].

Pindiprolu et al. [69] conducted a study to investigate the active targeting effectiveness of phenylboronic acid-modified niclosamide solid lipid nanomaterials (PBA-Niclo-SLN) for targeting TNBC cells that overexpress the SA receptor. The prepared formulation showed a significant increase in cell apoptosis, with a 21.3% increase compared to the control. Additionally, the cytotoxicity IC_50_ in MDA-MB231 cells was remarkably high at 7.3 µg/mL compared to the control. Moreover, PBA-Niclo-SLN demonstrated significant anticancer activity by reducing CD44 + /CD24- breast cancer stem cells from 88.1 to 27.6%. This suggests that PBA-Niclo-SLN has the potential to improve tumor bioavailability and reduce off-target side effects of niclosamide, effectively eradicating TNBCs [69].

The study by Scialla et al. [67] offered a synergistic effect using the magnetic resonance for diagnostic purposes and, iron oxide and doxorubicin (DOX) lipid-nanoparticles for chemotherapy purposes. On the other hand, Siddhartha et al. [68] and Pindiprolu et al. [69] showed the effect of SLN studies in improvement of specific targeting for antitumour activity in cells without a localizing effect [67–69]. The delivery of these medicines to specific tissues or cells enhances treatment outcomes while reducing adverse effects [21, 70]. Furthermore, micelles can be modified to include imaging agents like fluorophores, contrast agents, or radioisotopes. Micelles can therefore be used as imaging agents to visualise disease areas or to evaluate therapy responses in real time [70].

#### Micelles

Micelles have shown tremendous promise in drug delivery. The self-assembly of amphiphilic molecules in an aqueous medium results in the formation of small, spherical structures [31]. They can solubilize hydrophobic pharmaceuticals, increasing their stability and bioavailability. Micelles have the ability to encapsulate a wide variety of therapeutic agents, including chemotherapeutic drugs, therapeutic genes (siRNA), photothermal agents, and immunostimulants [70]. Micelle combination therapy in cancer research has the potential to have synergistic effects and enhance therapeutic outcomes. Personalised medicine is made possible by the adaptability of micelles, which enables one to modify their characteristics to meet the unique requirements of various patients. It is possible to achieve the best treatment results by adjusting the micelle's composition, size, and surface qualities in accordance with the unique patient features [70].

Wang et al. [71] developed and characterized a quantum dot (QD)-based micelle that was conjugated with an anti-epidermal growth factor receptor (EGFR) nanobody and loaded with the anticancer drug aminoflacone (AF) for targeted therapy and diagnosis of overexpressing cancers. They investigated the cellular uptake and cytotoxicity of these micelles in EGFR-overexpressing MDA-MB-468 triple-negative breast cancers (TNBCs). The in vivo results revealed high concentrations of micelles in the tumors compared to the control group without AF-encapsulated nontargeted micelles. The Nb-conjugated and AF-encapsulated QD-PLA-PEG micelles exhibit strong cytotoxic effects in EGFR-overexpressed TNBC. Moreover, no signs of systemic toxicity were observed in the mice, as evidenced by their constant body weight throughout the experiment. The proposed delivery system, a quantum dot-based conjugated micelle, shows great potential as an effective nanoplatform for EGFR-overexpressing cancers like TNBCs, offering the advantages of targeted therapy and reduced systemic toxicity [71].

A variety of fluorophores have been found to exhibit excellent cellular imaging capabilities. However, when used in vivo, they often suffer from poor solubility, leading to a short half-life. To overcome this challenge, Zhuang et al. [70] synthesized a DOX loaded mPEG-SS-Poly (AEMA-co-TBIS) (mPEATss) co-polymer to form multifunctional polymeric micelles that demonstrate aggregation-induced emission (AIE) activity for two-photon bioimaging. The study revealed that mPEATss micelles displayed exceptional AIE activity for two-photon cell imaging and deep tissue imaging. The DOX loaded mPEATss micelles showed significant anti-tumor effects, as evidenced by the reduction in tumor size from 1300 to 370 mm^3^ compared to the control group treated with saline water. Throughout the duration of the experiment, the mice's body weight remained constant, indicating that the micellar formulation was non-invasive and biocompatible. Both in vivo and ex vivo imaging, as well as in vitro studies, demonstrated the accumulation of micelles at the tumor site. This suggests that mPEATss micelles have the potential to be effective in treatment of TNBC, offering excellent anti-tumor efficacy with reduced off-targeting and effective bioimaging of cells and tissues [70].

In another study by Jin et al. [72] the authors synthesized cyclic arginine-glycine-aspartic acid (cRGD) peptide-decorated conjugated polymer (CP) nanomaterials. The resultant NPs were conjugated with poly (MEH-PPV) and characterization was done using a zeta sizer, transmission electron microscopy and a fluorimeter. The formulation was observed targeting capabilities of ανβ3 integrin-overexpressed in TNBC negligible cytotoxicity (0, 2, 4, and 6 nM) with the cell viability of 85%. Also, the novel formulation was reported to retain its fluorescent properties even after 30 days at above 70% [72].

The micelles studies above uniquely demonstrated reduction in cytotoxicity in healthy cells thus less adverse effects in vivo compared with the unbound drugs and an increase in cellular uptake in tumour cells thus offering a resounding activity as antitumor agents [65–72].

#### Dendrimers

Dendritic nanomaterials, which are nanosized (10–100 nm) particles with highly branched structures mimicking dendritic trees, are the main type of dendrites used in nanotechnology [21, 73]. Drugs or genes can be carried by dendritic nanomaterials and delivered to specific target sites in the body. The vast surface area made possible by their highly branching structure can be functionalized with different molecules to facilitate targeted drug delivery and improve the efficacy of therapy [74]. Dendritic nanomaterials can include imaging agents, making them suitable for use as contrast agents in MRI, CT, and fluorescence imaging, among other imaging methods. Dendritic nanomaterials contribute to nanomedicine by offering real-time imaging and monitoring of therapy responses by integrating therapeutic and imaging functions. The regulated release of therapies can be triggered by stimuli like pH or temperature using dendritic nanomaterials, which will improve drug release at the target site.

Torres-Perez et al. [75] conducted a study in which they synthesized and characterized Polyamidoamine dendrimers (PAMAM) loaded with methotrexate and D-glucose (OS-PAMAM-MTX-GLU) in the TNBC cell line, MDA-MB-231. The results demonstrated a remarkable 93.37% cytotoxic effect in MDA-MB-231 cells. The formulation of dendrimers exhibited a two-fold rapid and efficient delivery of a high payload of methotrexate to cancer cells compared to non-cancerous cells. These findings suggest that the OS-PAMAM-MTX-GLU delivery system holds potential as a targeted therapy for TNBC [75].

#### Quantum dots

**Fig. 6:**
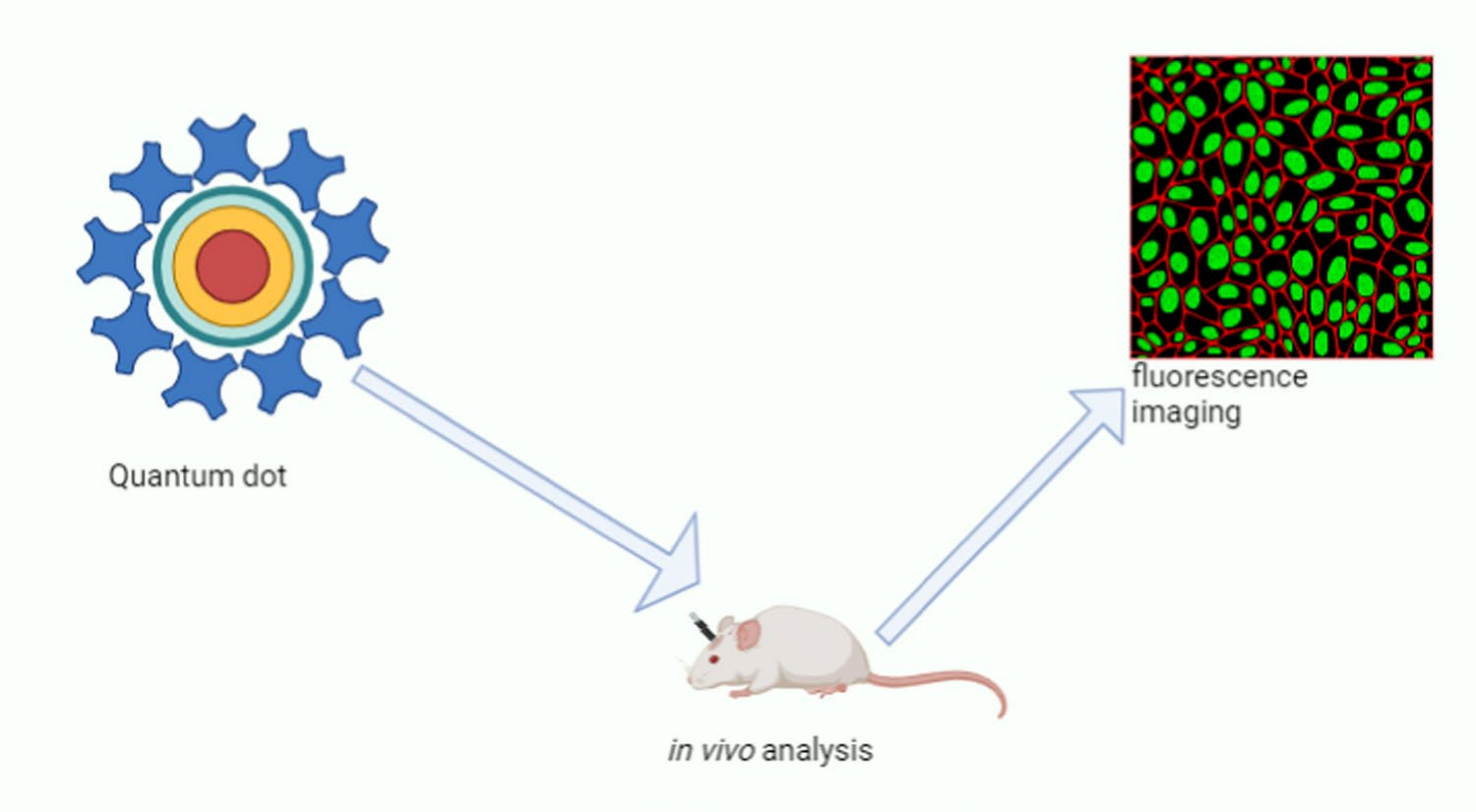
The structure and application of quantum dots [31, 77]

Quantum dots (QDs) have become adaptable nanomedicine tools. QDs depicted in Fig. 6 are semiconductor nanomaterials with distinctive optical characteristics. They are excellent candidates for applications in drug delivery due to their size-tunable emission and excellent photostability [31]. QDs can be used as carriers for therapeutic agents in drug delivery, allowing for targeted drug delivery. Because of their small size, they can successfully penetrate tissues and cellular barriers, delivering medications to the desired site and minimising off-target effects. QDs can also be applicable in both photodynamic and phototherapy where reactive oxygen species are generated upon light irradiation, destroying cancer cells through light-activated reactive species, and converting light into heat when irradiated, respectively [76].

In a study by Zhao et al. [76] a cancer cell membrane-coated biomimetic black phosphorus quantum dots (BBPQDs) were formulated and evaluated for tumor-targeted photothermal immunotherapy. The researchers proposed a combined strategy of photothermal therapy and anti-PD-L1 mediated immunotherapy for enhanced efficacy. The BBPQDs demonstrated a significant 34.90% increase in cell apoptosis and exhibited strong photothermal antitumor activity. In vivo biodistribution analysis revealed efficient tumor targeting with the formulation signals predominantly present in tumors compared to other organs. Moreover, BBPQDs effectively inhibited cancer cell recurrence and metastasis. This research introduces a novel therapeutic concept for treating TNBC, offering promising applications in the field [76].

**Fig. 7:**
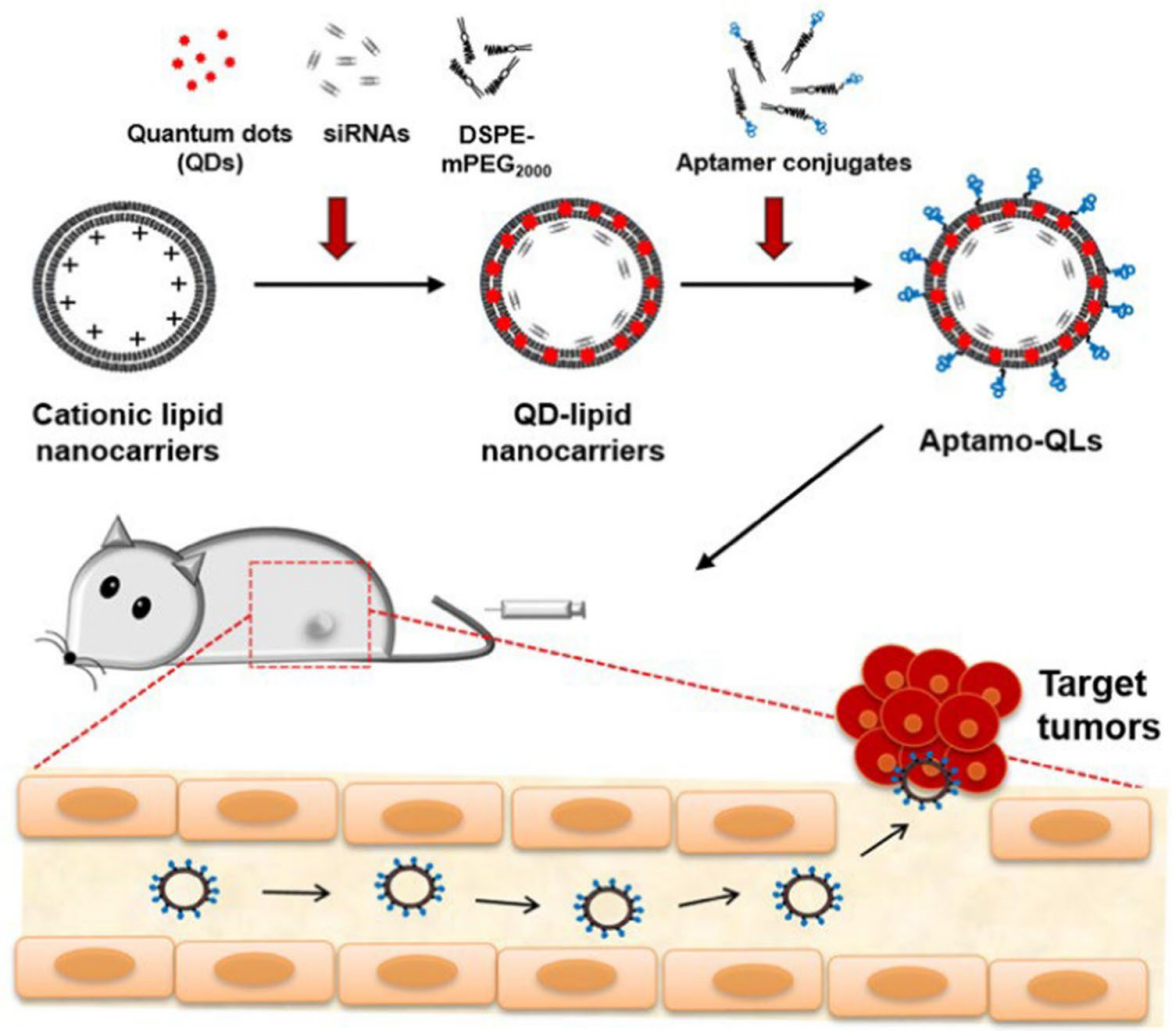
Depicts the formulation of theranostic strategy for RNAi gene therapy and fluorescence tumor imaging [58]

Developing carriers that encapsulate siRNA guided by aptamers has been challenging due to the similarity in their physicochemical properties. Kim et al. [58] formulated and characterized aptamer-coupled lipid nanocarriers encapsulating QDs and siRNAs for TNBC therapy as, demonstrating enhanced delivery to cancer cells, improved gene silencing, and tumor imaging as shown in Fig. 7. The combined therapy significantly inhibited tumor growth of 44.89% and metastasis, highlighting the potential of this delivery vehicle for RNA interference and fluorescence imaging in TNBCs using QDs [58].

Wang et al. [77] proved in their study that nanomaterials bound to biological molecules can target specific tumor cells, limiting the toxicity effects of chemotherapeutic agents to healthy cells. Quantum dot (containing indium phosphate and zinc sulfide) micelle nanomaterials were conjugated with the anti-epidermal growth factor receptor nanobody and subsequently loaded with the chemotherapeutic agent, aminoflavone (AF-encapsulated Nb-conjugated QD-PLA-PEG micelles). Quantum dots used in this study had high photostability, emitted near-infrared fluorescence and were imaged using optical imaging. AF-encapsulated Nb-conjugated QD-PLA-PEG micelles significantly induced tumor regression in TNBC cells. Further analysis of the results revealed that there was a notable increase in the uptake of the formulation as result of conjugation. Also, the increased fluoresce was observed in MDAMB-468 tumor-bearing mice after intravenous admission of the synthesised formulation [77].

Quantum dots are also garnering significant attention as their use in nanoplatforms is rising exponentially. Of interest are quantum dots with NIR fluorescence especially those with deep tissue penetration. An investigational study by Wang et al. [77] synthesised a quantum dot-based micelle using QD-PLA-PEG block copolymer polylactideb-poly(ethylene glycol) (PLA-PEG) and then conjugated using Anti-EGFR nanobodies, 7D12 Nbs while using aminoflavone as an anticancer agent. Analysis of the results revealed that there was a notable increase in the uptake of the formulation as result of conjugation. Also, the increased fluoresce was observed in MDAMB-468 tumor-bearing mice after intravenous admission of the synthesised formulation [77].

Another approach of interest is the encapsulating of the anticancer agent with an imaging compound and then conjugate with a protein. This approach was explored by Choi and Kim [31], using doxorubicin (DOX) as an anticancer agent, while diagnostic quantum dots (QDs) were used for imaging [31]. The encapsulated formulation was further conjugated with anti-epidermal growth factor receptor (EGFR) antibody. In vitro analysis was done using MDA-MB-231 and MDA-MB-453 breast cancer cell lines and the IC_50_ values of the DOX, biocompatible erythrocyte-derived nanomaterials and iEDNs-DOX were observed to be 0.02 μM, 0.18 μM, and 0.1 μM, respectively. The authors evaluated the biodistribution of the novel formulation and it was revealed a promising delivery strategy to the tumor sites with reduced accumulation in the liver. Also, the researchers assessed the anticancer activity of intravenously administered DOX alone, EDNs-DOX, and iEDNs-DOXin mice for 32 days. The outcome of the in vivo investigation demonstrated that the novel formulation significantly reduced tumor size from to 0.06 g as compared to other delivery systems.

The study by Zhao et al. [76], Wang et al. [77], Kim et al. [58] utilized membrane-coated biomimetic black phosphorus quantum dots, specific antibodies and siRNAs combinations that exhibited high photothermal conversion efficiency,improved biodistribution and effectively target specificity to tumour cells tumour homing [58, 76, 77].This again show the promising role of nanomaterials which can possibly change the current practice of medicine.

#### Carbon nanotubes

Carbon nanotubes (CNTs) are cylindrical structures primarily made up of carbon atoms arranged in a nanoscale tube-like pattern. As a result of their high surface area and flexible chemistry, CNTs enable excellent drug delivery to certain tissues or cells and have a high drug loading capacity [78]. Due to their high optical absorbance in the near-infrared range, which enables accurate imaging of biological structures and real-time monitoring of treatment responses, CNTs can be used in photothermal therapy, much like QDs. Carbon based nanoparticles have been reported to have stimuli response properties which are essential in TNBC management [76]. In bioimaging of TNBC, carbon nanoparticles are also promising due to their optical and chemical properties for non-invasive imaging. Furthermore, in clinical settings carbon-based nanoparticles have vindicated their versatility as imaging probes for breast cancer therapy.

Singhai et al. [79] synthesized multi-walled carbon nanotubes (MWCNTs) and functionalized them with Hyaluronic acid (HA) and α-Tocopheryl succinate (α-TOS). Loading these MWCNTs with Doxorubicin (Dox) resulted in the novel α-TOS-HA-MWCNTs/Dox conjugate. This formulation aimed to achieve enhanced cellular placement and therapeutic action against CD44 receptors overexpressing triple-negative breast cancer (TNBC) cells (MDA-MB-231). The outcome of the study demonstrated that α-TOS-HA-MWCNTs/Dox exhibited high cellular uptake compared to other MWCNTs formulations. Its anticancer efficacy was remarkable, showing significant growth inhibition (SRB assay; GI50; 0.810 ± 0.017; *p* < 0.001) and a high total apoptotic ratio (Annexin V/PI assay; 52.69 ± 4.86%; *p* < 0.005) in MDA-MB-231 cells. These findings suggest that α-TOS and HA can be synergistically and safely employed as tumor-targeted chemotherapy, highlighting the potential of the α-TOS-HA-MWCNTs/Dox conjugate for targeted therapy when tested using CD44 receptors overexpressing TNBC cells, offering a promising approach in breast cancer treatment [79].

In their study, Nabawi et al. [78] developed and characterized a formulation comprising sorafenib, carbon nanotubes, and folic acid for targeted delivery in TNBC. They investigated the in vivo pharmacokinetic properties and in vitro pharmacodynamics of the formulation. The in vitro studies showed increased apoptotic cell death, with elevated levels of caspase-3, caspase-8, and caspase-9 assay markers at 23.75 ng/ml, 5.38 ng/ml, and 47.76 ng/ml, respectively, along with a significant MTT cytotoxicity assay yielding a result of 2.91 ug/ml. Additionally, the formulation demonstrated enhanced bioavailability and a three-fold prolongation of the radioactive half-life. These promising results indicate the potential of this novel therapeutic strategy for TNBC treatment [78].

The above studies by Singhai et al. [79] and Nabawi et al. [78] demonstrated the use of carbon tubes in providing high treatment efficacy and specificity for TNBC. The studies showed an improvement in bioavailability, drug half-life and a very high growth inhibition effect thus rendering a potential treatment pathway for TNBC.

#### Gold nanomaterials

Gold nanomaterials (GNPs) are tiny particles of sizes ranging from 1 to 100 nm. They possess unique physical and chemical properties due to their size and shape, making them widely studied and used in various fields, including medicine, electronics, and environmental science [80]. Gold nanomaterials (GNPs) have shown significant potential in nanomedicine as shown in Table 2, which is the integration of therapy and diagnostics in a single platform. GNPs are used as versatile nanocarriers to deliver therapeutic agents such as drugs, genes (siRNA), photothermal agents, and immunostimulants to specific disease sites.

**Table 2:** Some of the reported studies using nanobased delivery systems in TNBC

Nano-delivery system	Structure	Targeting ligands	Targeting mechanism
Liposomes	Receptor mediated interaction directed by targeting ligands such as antibodies, peptides and aptamers on the surface	Folate ligands for folate receptor (FRα) targeting or antibodies against TNBC-specific biomarkers like HER2 or CD44	Passive targeting via enhanced permeability and retention (EPR) effect or active targeting which involves specific binding of targeting ligands to receptors overexpressed on TNBC cells, leading to enhanced cellular uptake and intracellular drug delivery [60, 62]
Solid Lipid Nanomaterials	Controlled drug release at target facilitated by temperature	Folate ligands or TNBC-specific antibodies	Like Liposomes use active and passive accumulation via the EPR effect and active through specific binding of ligands to TNBC cell surface receptors [67, 69]
Micelles	Encapsulated water insoluble agents in the hydrophobic core protects the active ingredient from update by the reticuloendothelial system	TNBC-specific biomarkers or receptors are preferred for enhanced tumor targeting	Capable of passive targeting through the EPR effect leading to accumulation in TNBC tissues [70–72]
Quantum dots	Targeting ligand are conjugated with the surfaces for precise imaging and targeted therapy	Antibodies, peptides, or aptamers for specific recognition of TNBC biomarkers or receptors. Ligands targeting TNBC-specific markers like HER2 or folate receptors can facilitate precise tumor targeting	Targeting ligands to bind to TNBC cells specifically, allowing for visualization and delivery of therapeutic agents to tumor sites [31, 58, 76]
Dendrimers	Have multivalent surface which can be functionalised with targeting ligands for selective binding to TNBC cells. For example, antibodies, peptides, aptamers, or small molecules	Folate ligands or TNBC-specific antibodies, are commonly used	Active targeting mechanisms, where targeting ligands facilitate specific interactions with TNBC cell surface receptors, leading to increased cellular uptake and intracellular drug delivery [61]
Carbon Nanomaterials	Biocompatible allowing for functionalisation with tumor-targeting ligands (antibodies, peptides, or aptamers) for specific target cell drug deliver and imaging	Folate ligands or TNBC-specific antibodies	Passive accumulation via the EPR effect and active targeting through specific interactions between targeting ligands and TNBC cell surface receptors, enabling efficient drug delivery to tumor sites [75]
Gold Nanomaterials	Functionalised with targeting ligands or coated with drug-loaded shells for targeting drug delivery and imaging. targeting ligands such as antibodies, peptides, aptamers, or small molecules for specific recognition and binding to TNBC cells	Ligands targeting TNBC-specific biomarkers or receptors are preferred for enhanced tumor targeting	They can be conjugated with targeting ligands to bind to TNBC cells specifically, allowing for visualization and delivery of therapeutic agents to tumor sites [80, 81]

Peng et al. [80] developed a nanocarrier called mesoporous magnetic gold "nanoclusters" (MMGNCs) to combine chemotherapy and photothermal therapy for tumor inhibition. In vitro cytotoxicity results showed that the co-therapy (chemo and photothermal therapy) was more effective in inhibiting tumor cell growth compared to MMGNCs combined with extra MF targeting. The IC_50_ of DOX-loaded MMGNCs in the co-therapy was 2.26 μg/ml, indicating enhanced efficacy [80].

Ruan et al. [81] developed and assessed a novel multistage system (G-AuNPs-DOX-RRGD) with active targeting and size-changeable properties to inhibit tumor growth and metastasis in mice with 4T1 xenografts. In vitro results revealed that G-AuNPs-DOX-RRGD could shrink from 185.9 to 71.2 nm after 24 h of incubation with MMP-2, and DOX release was pH-dependent. These findings suggest that the G-AuNPs-DOX-RRGD system exhibits excellent anti-tumor capacity due to the combined effects of RRGD targeting and the size-changeable with targeting ligands to selectively bind to specific receptors on diseased cells, enabling targeted drug delivery and imaging [81].

### Improving targeted therapy of nanoparticles on triple negative breast cancer

Nanoparticles have been investigated for targeted therapy of TNBC due to their ability to accumulate in tumor cells via CD44 receptor mediated endocytosis [82]. As previously discussed, nanomaterials have several advantages: reduced toxicity, improved biodistribution, improved pharmacokinetics, high accumulation in tumor sites, sustained drug release and longer half-life [82–84]. Smart nanomaterials have been employed in the delivery of TNBC therapeutic agents, unlike conventional NPs, their surface is modified in such a way that NPs achieve bio-responsive drug release, for example a disulfide linker that is pH-responsive can be used to conjugate a drug and a polymer [85, 86]. Upon stimulation by environmental factors such as pH, temperature, light, antibodies and enzymes, smart NPs are able to produce fast drug release in the tumor environment [86, 87]. Surface modification of mostly polymeric and liposomal nanomaterials through PEGylation has shown to reduce uptake of NPs by the reticuloendothelial system and increases their circulation time [88].

#### PEGylation of nanoparticles in TNBC therapy

Pegylated (PEG) doxetacel (DX)-loaded human serum albumin (HSA) NP bound to durvalumab (DVL) were prepared in a study by Yurt et al., (2023). HSA NP were employed in this study as they offer several benefits such as biocompatibility, reproducibility and easy synthesis. The drug loading efficiency of DX in HSA NPs was 91.8 ± 3.9%. The mean particle size of HSA-DTX@PEG-DVL was 130.4 ± 4 nm and zeta potential reading of − 30.1 ± 1 mV. Cytotoxicity study results revealed that the combination of DX and DVL in HSA-DTX@PEG-DVL NP exhibited high cytotoxicity in MDA-MB-231 TNBC cells. HSA-DTX@PEG-DVL NP were more effective in the early stage of apoptosis [88].

In a study by Wang et al. [71], PEG-grated chitosan copolymer NPs that are glutathione and pH-responsive were loaded with methotrexate (MTX) and magnolol (MAG). PEG-grated chitosan copolymer NPs interlinked with disulphide bond will degrade under glutathione, which is highly concentrated in the cytoplasm, thereby triggering the release of MTX which will exert synergistic antitumor effect with MAG. The drug release of MAG and MTX in simulated tumor-endosome microenvironment was 68.05 ± 3.27% and 35.86 ± 2.99%, respectively. The tumor survival rate of PEG-grated chitosan copolymer NPs loaded with MTX and MAG was 21.94 ± 1.43%. The synergistic effect of MTX and MAG and the use of NP attributed to the low tumor survival rate in comparison to MTX used alone, with a survival rate of 43.66 ± 1.77% [71].

The PEGylation of Docetaxel (DTX) for usage in TNBC MDA-MB231 cells was reported by Palma and associates. The nanoformulation was synthesized with DTX nanomaterials and hydroxypropyl-β-cyclodextrin via block copolymers of poly(ethylene glycol)-block-poly(ε − caprolactone) methyl ether were used as polymers. The results of the investigation showed that, when compared to the commercial formulation of DTX (Taxotere®), DTX-NPs were found to be effective as free DTX in suppressing the MDA-MB231 cells, even at low doses. In addition, the nanoformulation demonstrated better survival in a TNBC animal model and equivalent in vivo antitumor activity. This study therefore demonstrates that PEGylated biodegradable DTX-NPs improves their anticancer activity in TNBC [89].

A different study employing PLGA nanoparticles loaded with curcumin was reported by Prabhuraj et al. [89]. The nanoparticles were synthesized by conjugating several namely transferrin (Tf), hyaluronic acid (HA), and folic acid (FA), with polyethylene glycol (PEG) coatings. The TEM characterization results showed that that PEG was evenly coated on the surface of the PLGA nanoparticles, causing the size of the nanoparticles to grow from 85 nm (PLGA alone) to roughly 124 nm (PLGA-Cur-PEG-ligand nanoparticles). The results of the cell viability tests on MDA-MB-231 cells at an 80 μM drug concentration indicated that the viability for free Cur in PBS, PLGA-Cur, PLGA-Cur-PEG, PLGA-Cur-PEG-Tf, PLGA-Cur-PEG-HA, and PLGA-Cur-PEG-FA, respectively, steadily dropped from 92 to 41%, 32%, 39%, 19%, and 8% respectively [89].

#### Functionalisation using aptamers and antibodies

**Fig. 8:**
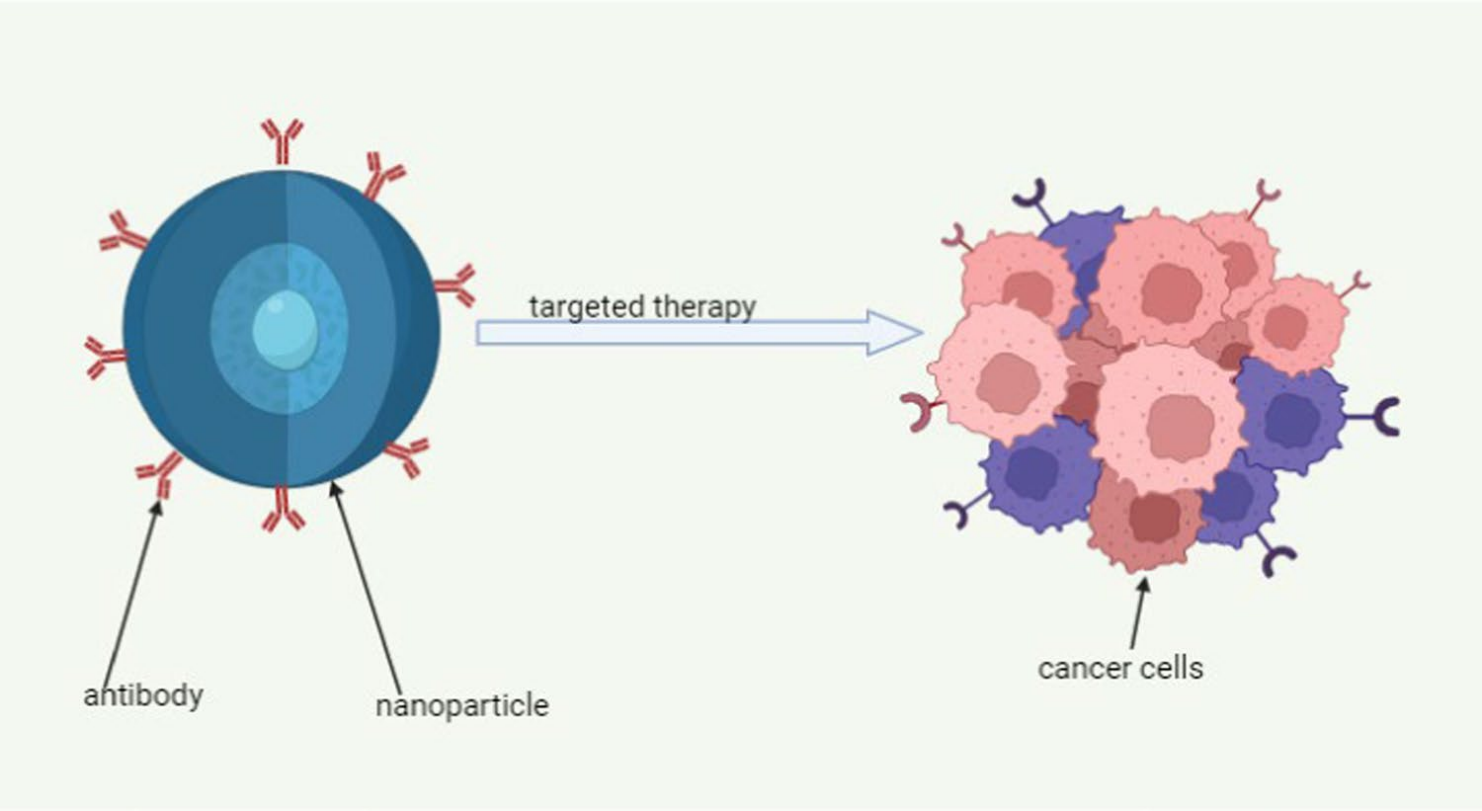
Shows the functionalisation of nanomaterials using antibodies to improve TNBC cell targeting [91, 92]

To enhance the targeted delivery of anticancer drugs for TNBC the pragmatic use of antibodies and aptamers has been linked with yield improved selectivity as depicted in Fig. 8**,** and efficacy. The precise delivery of anticancer drugs not only eliminates “off target” side effects but also allows for the development of safe and effective medicines. An example is the investigational work by Son et al. [90] anti-Trop2 antibody-conjugated nanoparticles (ST-NPs) as drug delivery systems for Doxorubicin (DOX). The findings of the study showed that DOX encapsulated in ST-NPs (DOX-ST-NPs) exhibited a swift release of DOX when exposed to 10 mM glutathione (GSH). However, the release of DOX was notably delayed under physiological conditions (PBS, pH 7.4). Additionally, the use of confocal microscopic images and flow cytometry analysis demonstrated that DOX-ST-NPs were specifically internalized by MDA-MB-231 cells, which serve as a model for Trop2-expressing TNBC cells [90].

Valcourt and colleagues [93] developed a drug delivery system using poly (lactic-co-glycolic acid) nanoparticles functionalised with Notch-1 antibodies, resulting in N1-ABT-NPs. The incorporation of antibodies enabled specific binding to TNBC cells and suppression of Notch signaling. The study demonstrated that the formulated N1-ABT-NPs selectively targeted TNBC cells over noncancerous breast epithelial cells, effectively regulating Bcl-2 and Notch signaling to induce cell death. Moreover, the researchers found that N1-ABT-NPs could accumulate in subcutaneous TNBC xenograft tumors in mice after systemic administration, leading to reduced tumor burden and prolonged animal survival [93].

Mariadoss and coworkers [94] prepared a p-Coumaric acid-loaded aptamer (ligand) conjugated starch nanoparticles (Apt-p-CA-AStNPs) for the treatment of TNBC. The outcome of the study showed that Apt-pCA-AStNPs exhibited increased cytotoxicity in MDA-MB-231 cells through specific targeting of nucleolin. Furthermore, the administration of Apt-pCA-AStNPs induced cell death in MDA-MB-231 cells by modulating the process of apoptosis. The findings of this study underscore the fact that the application of aptamer-based drug delivery systems presents a hopeful avenue for enhancing cancer therapy [94].

Camorani et al. [95] recently developed the aptamer delivery system for TNBC therapy. The authors successfully developed drug delivery systems using programmed cell death-ligand 1 (PD-L1) to enhance the targeting of targeting (TNBC) cells. The nanoformulation was prepared utilizing Poly(lactic-co-glycolic)-block-polyethylene glycol (PLGA-b-PEG) loaded with (PD-L1) and further evaluated for its toxicity and selectivity. The cytotoxicity studies demonstrated that the aptamer formulation exhibited an increased selectivity and this was ascribed to improved binding and selectivity resulting in the targeted release of siPD-L1 in TNBC cells [95].

#### Formulation of chemotherapeutic agents as nanoparticles

Chemotherapeutic drugs are known to be cytotoxic, have poor solubility, low tumor selectivity and have devastating side effects. To combat the drawbacks connected with the utilisation of chemotherapeutic agents, the formulation of anticancer drugs as nanoparticles may significantly improve the efficacy of these drugs and reduce their side effects. Over the past decade, studies as depicted in Table 3 have been reported to improve the chemotherapeutic agents especially in TNBC. Of interest is the study by Liu et al. [47] aimed to investigate the potential of tmTNF-α monoclonal antibody (mAb)-conjugated paclitaxel (PTX) nanoparticles (NPs) as nanocarriers for targeted drug delivery. In this study, tmTNF-α monoclonal antibody (mAb) were then conjugated for functionalisation purposes with paclitaxel (PTX) nanoparticles (NPs) were used. The results of the study demonstrated that the prepared formulation in comparison to the control groups, the growth of tumors in human MDA-MB-231 xenograft mice was significantly suppressed by the tmTNF-α mAb-PTX NPs. The mechanism of the nanoformulation in inhibiting cancer cell growth was attributed to anti-tumor effects by enhancing apoptosis and modulating various signalling pathways, including mitogen-activated protein kinase (MAPK) [47].

**Table 3:** Some of the reported studies on the formulation nanoparticle-based systems using anticancer drugs in TNBC

Chemotherapeutic agent	Nanoformulation	Cell line	Role
Doxil	Irna-loadable lbl	BALB/c mice	Improved drug delivery and enhanced anticancer activity [96]
Abraxane	Albumin nanoparticle	SUM149 cells	Enhanced drug efficacy [97]
Talazoparib	Talazoparib-slns	HCC1937	Increased the therapeutic efficacy of talazoparib [98]
Docetaxel	PLGA-docetaxel nanoparticles	MD-MBA-231 cells	Increased anticancer activity [99]
Paclitaxel-	Paclitaxel-loaded poly(D,L-lactide-co-glycolide) nanoparticles	MDA-MB-231-LM2	Improved cell targeting [100]
Doxorubicin (DOX) and mitomycin C (MMC)	Irgd-TPLN with coloaded doxorubicin (DOX) and mitomycin C (MMC) (irgd-DMTPLN)	Human MDA-MB 231-luc-D3H2LN and MDA-MB-468	Reduced the metastatic burden an increased host median survival [43]
Doxorubicin (DOX) and mitomycin C (MMC)	Arg-Gly-Asp peptide (RGD)-conjugated, doxorubicin (DOX) and mitomycin C (MMC) co-loaded polymer-lipid hybrid nanoparticles (RGD-DMPLN)	MDA-MB-231	Significantly inhibited the progression of lung metastases of TNBC and increased host cell survival [101]
Bortezomib	Bortezomib encapsulated nanoparticles	MDA-MB-468 and HCC1937	Increased cell proliferation and apoptosis induction [102]
Paclitaxel	(lactic-co-glycolic acid) (PLGA) nanoparticles embedded with paclitaxel and coated with hyaluronic acid (HA-PTX-PLGA)	MDA-MB-231	Increased the potency of PTX [103]
Paclitaxel	Paclitaxel amino lipid (PAL) derived nanoparticles	MDA-MB-231 cells	Significantly reduced tumor growth [43]

Another study reported the preparation rod-shaped PLGA-docetaxel nanoparticles nanoparticles to encapsulate docetaxel. To prepare this formulation of nanoparticles Particle Replication in Nonwetting Templates (PRINT) technique was used. Evaluation of the pharmacokinetic profile revealed that the synthesized PLGA-docetaxel nanoparticles demonstrated an increase in the docetaxel circulation time as compared to the clinical formulation of docetaxel, Taxotere. Also, the authors highlighted that the nanoformulation significantly inhibited the growth of tumor cells [99].

In another study by Cano-Cortes et al. [25] trifunctionilsed a NP by linking it with an anticancer agent DOX, a dye (CY7) and a homing peptide (CRGDK). In this study DOX was used an anticancer agent, while the dye was used for bioimaging, and the peptide was used to target neuropilin-1 (Nrp-1) receptor. The synthesised nanoparticles were characterised by use of the DLS and the Zeta potential, Transmission Electron Microscopy, and atomic force microscopy (AFM). The nanoformulation also exhibited efficient targeted therapy and commendable imaging properties [25].

## Recommendations in practice

Recent advances in research show that new approaches for the treatment of Triple Negative Breast Cancer (TNBC) are required due to the ineffectiveness of some of the current treatment approaches [81]. Polymeric nanoparticles, metallic nanoparticles, dendrimers, liposomes, quantum dots, carbon nanotubes, layer by layer nanoparticles, and other types of nanoparticles (NPs) are deemed to be promising approaches in the treatment of TNBC [31, 104]. Nanoparticles are recommended for the treatment of TNBC because of their target specific multifunctional characteristics. Nanoparticles can be an excellent strategy for TNBC treatment if they are designed appropriately. Triple negative breast cancer (TNBC) is a very challenging subtype of breast cancer with a poor prognosis [31]. Quantum dots have been proposed for the treatment of TNBC due to their unique properties such as symmetric emission band, long fluorescence lifetime, and strong photostability. They offer advantages of higher sensitivity and more accurate quantitative analyses in TNBC treatment [31]. Black phosphorus quantum dots (BPQDs) are specifically recommended for their effective photothermal effects and faster clearance. However, BPQDs have limitations including instability and poor targeting ability. Recent studies have shown that coating BPQDs with cancer cell membranes can enhance their stability. These cancer cell membranes coated biomimetic BPQDs (BBPQDs) have demonstrated superior targeting capabilities towards TNBC cells compared to BPQDs. Furthermore, BBPQDs have exhibited enhanced direct photothermal cytotoxic effects and indirect antitumor activity through dendritic cell maturation [31].

Black phosphorus quantum dots (BPQDs) are specifically recommended for their effective photothermal effects and faster clearance. However, BPQDs have limitations including instability and poor targeting ability. Recent studies have shown that coating BPQDs with cancer cell membranes can enhance their stability. These cancer cell membranes coated biomimetic BPQDs (BBPQDs) have demonstrated superior targeting capabilities towards TNBC cells compared to BPQDs. Furthermore, BBPQDs have exhibited enhanced direct photothermal cytotoxic effects and indirect antitumor activity through dendritic cell maturation [31].

Carbon nanotubes are carbon allotropes with a cylindrical nanostructure, and they are recommended in practice because of their large surface areas, rich surface chemical functionalities, high penetrating capability, and size stability. Carbon nanotubes (CNTs) are recommended for use as vectors to deliver anticancer drugs and for photodynamic therapy (PDT). Studies show that collagen gels combined with MWCNTs can restrict cell contraction, invasion, viability, MMP-9 expression, and the migration of TNBC cells. Some studies show that the systemic administration of targeted SWCNTs exhibits remarkable tumor accumulation and photoacoustic imaging ability in the mouse model [31].

Liposomes are recommended in practice because they resemble biological membranes and consist of a phospholipid bilayer. These amphipathic, phospholipid-based NPs produce a co-delivery system that can carry both hydrophilic and hydrophobic medicines [105]. Liposomes are a promising nanocarrier for site-specific targeting and sustained drug release due to their non-toxic nature, biocompatibility, biodegradability, structural flexibility, size, and the membrane permeability of their lipid layers [105]. However, the medicinal uses of liposomes may be restricted by lipid oxidation, low water solubility, short half-life, and potential drug leakage. A targeting agent that can prolong drug release at the target site and reduce adverse effects could be added to liposomal formulations to increase their efficacy [106].

Metallic nanocarriers are recommended for routine use due to their inherent advantages. These advantages include their simple synthesis, variable size, charge, and shape, high surface-to-volume ratio, ease of surface modification, and thermal ablation [107]. Metallic NPs have a higher density and are more easily absorbed by cells than non-metallic formulations of comparable size, making them advantageous for targeting inside cancer cells. Metallic NPs also possess distinct optical properties that enable them to absorb light energy and facilitate tumor obliteration in a process known as photothermal treatment.

Dendrimers consist of a core inner and outer shell, and they are hyper-branched, three-dimensional, symmetric, spherical, and nanosized synthetic structures [107]. They offer a potential to conjugate high molecular weight hydrophilic agents via host–guest interactions or hydrophobic pharmaceutical agents through covalent conjugation. Various types of dendrimers exist, such as poly(etherhydroxylamine), poly(esteramine), polyamidoamine (PAMAM), poly(propyleneimine) (PPI), poly-l-lysine, melamine, and polyglycerol (PG). Dendrimers with intricate three-dimensional architectures are commonly used as pharmaceutical carriers due to advantages such as high aqueous solubility, hyperbranching, structural uniformity, accurate molecular weight, well-defined globular structures, permeability through biological membrane, multi-valency, chemical composition variability, and biocompatibility [107].

The target specificity and therapeutic effects of the drugs/NPs are enhanced through the utilization of monoclonal antibodies as targeting moieties conjugated to drugs (ADC) or NPs (Ab-NP) [105]. Therapeutic intervention is carried out following the accurate diagnosis of TNBC, and considering additional elements such as the metastatic nature, drug sensitivity/resistance, recurrence, and poor prognosis [21]. In TNBC, breast conservation therapy (BCT) is the initial option and an effort to prevent mastectomy. However, the high rate of tumor recurrence even after radiation therapy (RT) compels doctors to recommend mastectomy in addition to radiotherapy for patients [21]. Due to the absence of HER2 and hormonal ER and PR receptors, hormonal therapy that is effective in other subtypes of breast cancer is not applicable to TNBC, necessitating chemotherapy, which is presently the backbone of systemic treatment [21].

The treatment of TNBC is very difficult due to the increased risk of metastasis. High concentrations of cytotoxic medicines administered repeatedly during chemotherapy cycles kill nearby healthy cells in addition to cancer cells. Drug delivery methods based on nanotechnology are a viable solution to avoid non-specific targeting, cytotoxicity, and harmful adverse effects. Recent developments in nanotechnology allow for the selective targeting of cancer cells, as well as the elimination of the cytotoxicity of medicines to other organs. Since conventional therapies have many limitations such as rapid drug clearance and limited targeting, it is important to use nanotechnology in TNBC. To date, no targeted therapy has been developed which can effectively treat triple negative breast cancer [21].

## Conclusion and future aspects

TNBC is one of the most aggressive and increasing growing concern globally. The current treatment strategies unfortunately have limited applicability in most TNBC cases. To address the increasing case of TNBC it is imperative that other approaches be considered to circumvent the current global concern. Nanotechnology using nanoparticles offers a promising strategy to treat TNBC. To date, a notable number of studies have highlighted the role of nanoparticles in TNBC. Nanoparticles induce apoptosis, reduce side effects associated with chemotherapy drugs, enhance drug entrapment efficiency and ultimately bioavailability. These advantages associated with the use of nanoparticles in TNBC if thoroughly explored may significantly help to mitigate the challenges linked to TNBC therapy. Although a notable number of studies have been conducted on TNBC research, there are still gaps in the literature that require further exploration.

TNBC has been associated with various molecular subtypes and it is generally difficult to treat due to the lack of expression of ERs, PRs, and HER2. The synthesis of nanomaterials using both the bottom up or the top-down methods often involves the use of toxic solvents and reagents. Unfortunately, the synthesis stages and work-ups do not always eliminate all the toxic constituents from the final products. Consequently, the use of nanomaterials comes with limitations specifically linked to immunogenicity, biodegradability and toxicity. Some nanomaterial formulations significantly lack in their cell targeting abilities and this often results in altered pharmacokinetic profile of the drug. Also increased anticancer activity is mostly related to small particle size and in some cases the smaller the particle sizes the greater the toxicity. As a result, designing an ideal formulation with minimal side effects may prove to be challenging. Furthermore, the stochastic nature of ligand and receptor interactions which is predominantly used in targeted delivery remains a general source of uncertainty and ultimately affect the distribution of drugs.

Nanomaterials, like any medicine, can potentially induce toxicity and immune responses due to their interaction with biological systems. For instance, cationic lipids in liposomes and solid-lipid nanoparticles (SLNs) may cause toxicity or have limited drug-loading capacities. Another growing concern is that nanocarriers can be immunogenic and may be rapidly excreted from the body, reducing their effectiveness. SLNs have low drug-loading capacities and complex colloidal structures, while cationic lipids in liposomes can cause toxicity and suffer from rapid degradation. The optimal delivery to tumor sites remains challenging, with nanoparticles often cleared rapidly by the reticuloendothelial system (RES). To date, well-defined pharmacokinetic profiles of nanoformulations are essential to ensure the effective and safe application of nanomaterials in therapy. Parameters such as the zeta potential of nanoparticles affect treatment efficiency, with electronegativity preventing nonspecific cell targeting and reducing cytotoxicity. Charge-reversal nanoparticles can improve targeted accumulation and permeability.

Most nanomaterials have complex manufacturing processes and generally have poor stability and reproducibility, which consequently hinders scalability and clinical translation of nanotherapeutics. The reproducibility and scalability of nanoparticle manufacturing must be addressed to ensure they can be produced at a larger scale. Most nanoparticles tend to aggregate, affecting their properties and efficacy. Therefore, long-term stability needs to be evaluated to ensure they maintain their properties over time. The complex nature of nanomaterials poses challenges for regulatory approval. The regulatory landscape for nanoparticles is still evolving, with no specific guidelines for their use in clinical applications, creating uncertainty for researchers and companies.

The long-term impacts of nanomaterials on human health and the environment are still largely unknown. Although used to reduce immune recognition, PEG can induce blood coagulation and cellular agglutination. A significant proportion of healthy individuals possess antibodies against PEG, leading to expedited removal from the body. Major ethical concerns arise from the risks to participants in clinical trials, including issues related to germline gene editing. The advent of CRISPR technology further raises apprehensions about the ethical implications of genetic modifications.

Pharmaceutical ingenuity is needed to improve the formulation of targeted nanoparticles to improve specificity, thus minimizing side effects. The improvement of encapsulation efficiency of nanoparticles is another trajectory that should be explored to enhance drug delivery. Developing smart nanoparticles highly sensitive to tumor microenvironment changes to ensure targeted drug release. In TNBC therapy, bioimaging is pivotal, and incorporating imaging agents and stimuli-responsive elements to monitor treatment efficacy and optimize drug release kinetics. Nanoparticle-facilitated Immunotherapy is another strategy of combining nanoparticles with immunotherapy to overcome the immunosuppressive nature of the TME and improve therapeutic outcomes. Using nanoparticles to deliver inducers of immunogenic cell death (ICD), enhancing the activation of dendritic cells and reversal of immunosuppressive cells. Facilitate the translation of nanoparticle-based therapies from preclinical studies to clinical applications, focusing on efficacy across different TNBC subtypes. The art of surface modification is another lacking strategy. Through the use of biocompatible polymers to modify the surface of nanoparticles, reducing immune recognition and clearance by the reticuloendothelial system using nanoparticles to activate innate immune cells to enhance antitumor immunity. Designing nanoparticles that combine therapeutic agents with imaging capabilities for simultaneous diagnosis and treatment (theranostics). Researchers should strive to develop standardized protocols for nanoparticle synthesis to ensure consistency and scalability. manufacturing, and evaluating their long-term stability to ensure consistent efficacy. By addressing the reproducibility and scalability of nanoparticle manufacturing and evaluating their long-term stability to ensure consistent efficacy.

## Data Availability

No datasets were generated or analysed during the current study.
